# IAServ: An Intelligent Home Care Web Services Platform in a Cloud for Aging-in-Place

**DOI:** 10.3390/ijerph10116106

**Published:** 2013-11-12

**Authors:** Chuan-Jun Su, Chang-Yu Chiang

**Affiliations:** Department of Industrial Engineering & Management, Yuan Ze University, No. 135, Yuan-Tung Rd., Chung-Li City, Taoyuan 320, Taiwan; E-Mail: s978905@gmail.com

**Keywords:** ubiquitous computing, healthcare, aging-in-place, web service, cloud computing, ontology

## Abstract

As the elderly population has been rapidly expanding and the core tax-paying population has been shrinking, the need for adequate elderly health and housing services continues to grow while the resources to provide such services are becoming increasingly scarce. Thus, increasing the efficiency of the delivery of healthcare services through the use of modern technology is a pressing issue. The seamless integration of such enabling technologies as ontology, intelligent agents, web services, and cloud computing is transforming healthcare from hospital-based treatments to home-based self-care and preventive care. A ubiquitous healthcare platform based on this technological integration, which synergizes service providers with patients’ needs to be developed to provide personalized healthcare services at the right time, in the right place, and the right manner. This paper presents the development and overall architecture of IAServ (the Intelligent Aging-in-place Home care Web Services Platform) to provide personalized healthcare service ubiquitously in a cloud computing setting to support the most desirable and cost-efficient method of care for the aged-aging in place. The IAServ is expected to offer intelligent, pervasive, accurate and contextually-aware personal care services. Architecturally the implemented IAServ leverages web services and cloud computing to provide economic, scalable, and robust healthcare services over the Internet.

## 1. Introduction

Rapid advances in medicine and technology are leading to marked increases in human longevity. Combined with declining birth rates, societies around the world are now facing challenges associated with rapidly aging populations. A recent report from Taiwan’s Department of Health shows that nearly 90% of senior citizens in Taiwan have at least one chronic disease, while over half suffer from three or more chronic diseases. Many of these people are incapable of living independently, and their requirements for care will only continue to increase. These issues are raising broader concerns about the overall care and quality of life of the elderly.

Home care is conceived of as the integration of medical, social and familial resources to enable the integral care of elders in their own residences [1]. It emphasizes the concept of “aging-in-place” in which a range of products, services and conveniences are used to enable patients to remain in their own homes, even as their circumstances change [2]. The practice of aging-in-place would have the significant advantages of supporting elders’ feelings of dignity, quality of life and independence. Today, however, the current connections between elderly health and housing are tenuous at best. As a result, while aging in place is the most desirable and cost-efficient method of aging, it is difficult to implement, even under the most ideal conditions [3].

The goal of healthcare in home is to improve health outcomes in the population and to improve service personalization, while reducing inequalities in both health outcomes and responsiveness. The healthcare needs of the population should be met with the best possible quantity and quality of services produced at minimum costs. Information technology (IT) can be used to enhance the delivery of healthcare services and to improve quality of life. In this research, we aim to seamlessly integrate such enabling technologies as ontology, intelligent agents, web services, and cloud computing for establishing the Intelligent Aging-in-place Home care Web Services (IAServ) platform to provide ubiquitous, personalized healthcare services.

In the context of healthcare, virtually no cases have identical presentations, and every patient has a unique history and disease manifestation. A robust methodology is therefore needed for the provision of personalized healthcare services, which takes into account the health status and various contexts of each patient [4,5]. To provide personalized health care services, ontology and rules of inference are utilized in IAServ to convert low-level context to high-level context. Ontological design used in IAServ furnishes an accurate formalization of healthcare goals for responding to the needs of health care providers and patients by directing and addressing their current aims. Personalization in IAServ is achieved by compiling individual versions of a personal profile, which derives the action plan from personal agents in a multi-agent environment. The dynamic aspects of health care environments, such as the rapid growth in domain knowledge and constant change in patient conditions, are addressed using the Maintenance Agent.

IAServe is platform- and language-independent, and was built on the Representational State Transfer (REST) architecture [6,7], which is a simple set of principles to connect applications in a style native to the Web. REST web services in the IAServ provide an abstraction for publishing information and giving remote access to the data repository. It regards the information or data as resources, each of which is referenced with a simple Uniform Resource Identifier (URI). Using REST technology, IAServ is able to hide the complexity of its Service Oriented Architecture (SOA) [8,9] and to interoperate other applications via a simple URI.

The development of IAServ aims to improve the quality of aging-in-place, increase the proactivity of healthcare services, and reduce the overall cost of providing such services. An ontology that allows for the formalization of healthcare goals is used to create a personalized care plan for each patient. The Web Services furnish various applications relevant to the provision of care activities. Mobile Agents offer contextually-aware, timely and accurate information for the provision of quality care, with care and data recorded automatically.

Section 2 of this paper reviews related work to discuss the development of Ambient Intelligence (AmI) and personalized services. The section also presents a discussion of IAServ which combines Web services, cloud computing and ontology development. Section 3 presents the system design and architecture of the IAServ. Section 4 covers implementation details and demonstrates usage scenarios. Finally, conclusions and suggestions for future work are made in Section 5.

## 2. Related Work

### 2.1. Ambient Intelligence (AmI)

The early developments in AmI took place at Philips, a Dutch electronics conglomerate. The board of directors commissioned a series of internal workshops to investigate different scenarios that would transform the high-volume consumer electronics industry from “fragmented with features” into a post-modern world where user-friendly devices would support ubiquitous information, communication and entertainment [10].

AmI refers to a vision of the future in which people are empowered by an electronic environment that is aware of their presence, and is sensitive and responsive to their needs. It aims at improving quality of life by creating the desired atmosphere and functionality via intelligent, personalized interconnected systems and services [11]. AmI implies a seamless environment of computing, advanced networking technology and specific interfaces. It is aware of the special characteristics of human presence and personality, addresses human needs and is capable of responding intelligently to spoken or gestured commands, and can even engage in intelligent dialogue with the user [12].

A typical context of applying ambient intelligence is smart home environment [13]. Wireless communication technologies can be used to connect home appliances with a specified control unit, which acts as a middle-ware mechanism to enable the remote control and monitoring of appliances through mobile devices. AmI is built on the concept of contextual-awareness, which raises the possibility of a new generation of interactive applications and systems. However, the development and deployment of interactive systems are ultimately determined by context [14].

Schilit and Theimer [15] defined contextual-awareness in the domain of ubiquitous computing as being “the ability of a mobile user’s applications to discover and react to changes in the environment they are situated in.” Perhaps due to technical limitations, the actual contextual information that Schilit and Theimer used in their ActiveMap Service was limited to location only. They suggested that future contextually-aware applications should include much more than geographic information, a suggestion that numerous researchers have attempted in building upon Barkhuus’s research [16]. The Context Broker Architecture (CoBrA) is an agent-based architecture supporting context-aware systems in smart spaces (e.g., intelligent meeting rooms, smart homes, smart vehicles, *etc.*) [17]. Central to this architecture is an intelligent agent called the context broker that maintains a shared contextual model on the behalf of a community of agents, services, and devices within the space and protects user privacy by enforcing user-defined policy rules.

### 2.2. AmI Applied in Care Services

Context-aware computing systems and methods are described, along with particular embodiments of location aware systems and methods. In the described embodiments, hierarchical tree structures are used to ascertain a device context or location [18]. Contextual awareness is not a new concept, but technologies (e.g., wireless technologies, mobile tools, wearable instruments, intelligent artifacts, handheld devices, *etc.*) are only now becoming available to support the development of contextually aware applications. Such technologies could help health care professionals manage tasks while increasing the quality of patient care [19].

Su and Chiang proposed an “Ambient Intelligent Community Care Platform” (AICCP) by applying radio-frequency identification (RFID) and Mobile Agent technologies to enable care givers and communities to offer pervasive and contextually-aware care services [20]. RFID allows care-givers to easily locate the patients, while Mobile Agents furnish contextually-aware, timely and accurate information for the provision of quality care, with care and treatment data recorded automatically. Coronato *et al.* presented a semantic location service to locate active mobile objects such as mobile devices and RFID-tagged objects in smart and intelligent environments [21]. The location service is constructed based on the ontologies and rules, which specify a uniform, well-defined and unambiguous model for the location information, but not by use of particular positioning system. Fraile *et al.* presented a hybrid Multi-Agent architecture, named HoCa, for the control and supervision of dependent environments [22]. The HoCa architecture provided the basic idea of incorporating an alert management system based on Short Message Service (SMS) and Multimedia Messaging Service (MMS) technologies and context control system based on Java Card and RFID technologies. Fenza *et al.* developed an integrated environment to provide personalized healthcare services based on agents, sensor networks, and ontologies. The context recognition, service matchmaking and brokerage activities are achieved through the use of agent technology [23,24]. The IAServ proposed in this paper integrates several novel concepts, (e.g., cloud computing, web services, *etc.*), to further increase the robustness of care service provision.

### 2.3. Profiles and Personalized Services

The user profile is the data instance of a user model that is applied to adaptive interactive systems. It also acts as the key component used to provide personalized services or information in the personalization system [25,26]. Eslami *et al.* proposed an effective service tailoring processes and an architecture to personalize homecare services according to the needs of the individual patient. In the proposed approach, the tailoring process is divided into six steps to configure the personalized services from the user profile [27]. Chang *et al.* developed a personalized service recommendation system (PSRS) in a home-care environment. The PSRS can provide appropriate services based on the user’s preferences and habits recorded in the user profile. Through the user profile, the system will be able to automatically launch safety alerts, recommended services and healthcare services in the patient’s house [28].

The feasible solution to generating personalized services from the user profile entails two processes: generating goal activities from the user profile and defining the services which can fulfill those activities. In generating the goal activities, a “goal ontology” is created to represent the relationship between the user profile and goal activities. In defining the services, a “task ontology” is created to specify the relationship between the goal activities and the services. This solution has been widely used in many studies [26,27,28]. Here, this solution is also cited to support the configuration of personalized services configuration.

### 2.4. Ontology-Based Approach for Knowledge Intensive Applications

An ontology describes the concepts and relationships that are important in a particular domain, providing a vocabulary for that domain as well as a computerized specification of the meaning of terms used in the vocabulary. The aim of an ontology is to generically formalize domain knowledge and provide a common understanding of a domain, which may be used and shared by applications and groups. It has been widely adopted in the business and scientific communities as a way to share, reuse and process domain knowledge [29]. Ontologies are also central to many applications in fields including information management, systems integration and semantic web services [30]. Many studies have also demonstrated that ontology is essential for the development of knowledge-oriented systems.

In the critical medicine and health care fields, ontologies are valued and broadly applied to building knowledge intensive services, applications, and systems. Ontology-based computational models have been proposed to facilitate effective management and acquisition of medical knowledge [31,32], and to serve as the foundation for medical diagnosis and treatment [33,34,35]. The diagnosis and treatment systems are constructed based on ontologies and rules. The ontologies define the medical knowledge models, such as the physiological symptoms, disease features, and treatments. The rules specify the relationships between symptoms, disease, and treatments to determine the diagnosis and period of treatment. Moreover, Su and Peng addressed the ontological and epistemological issues of information services through the example of OntoRis, an ontology-based rehabilitation service designed to assist patients in acquiring actionable knowledge about his/her prescribed rehabilitation, and to expedite recovery by providing suggestions and advice drawn from evidence-based medicine [36].

### 2.5. Web Service Approach in Information Integration

Techniques have been described for facilitating the use of invocable services by configurable applications. In at least some situations, the invocable services are Web services (WSes) or other network-accessible services that are made available by providers of the services for use by others in exchange for fees defined by the service providers [37]. Such techniques may be used, for example, in conjunction with an electronic WS marketplace via which third-party WS providers make their WSes available to third-party WS consumers who locate and purchase access to those WSes, thus allowing a user to dynamically create a new composite WS based on one or more WSes available from other WS providers and that reflects any constraints of the WS marketplace, with the composite WS available for use by other WS consumers [38].

Recent Web 2.0 developments show how easy it can be to dynamically link or compose IT components to achieve the original SOA goals of flexibility, reusability, or reduction by relatively simple means [39,40]. At this point the idea of representational state transfer (REST) [41] is often used in a Resource-oriented Architecture (ROA), which uses only Hypertext Transfer Protocol (HTTP) methods such as POST, GET, PUT and DELETE as actions. REST specifies a collection of architectural principles defining how data resources are represented and addressed. When the REST architectural principles are applied, as a whole, they provide enhanced scalability, interface generality, and deployment independence, while reducing interaction latency and encapsulating legacy systems [41]. Given these advantages and characteristics, REST WSes are broadly applied in information system integration in research fields including smart homes [42], e-Learning [43] and engineering collaboration [44], *etc.*

## 3. IAServ Design and Architecture

**Figure 1:**
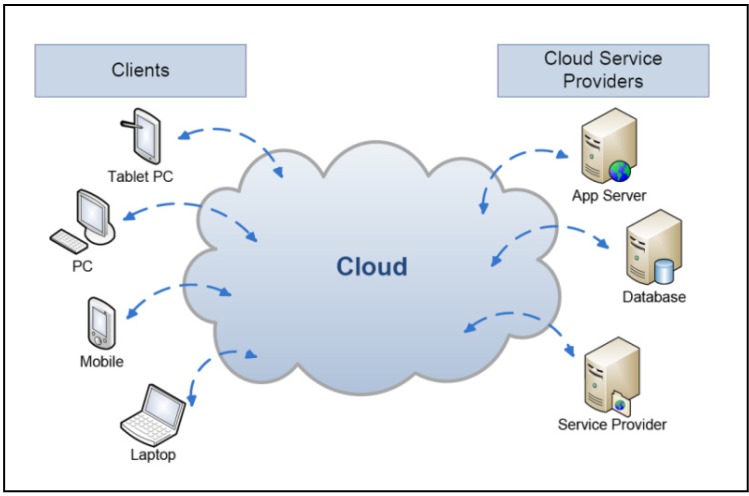
The concept of cloud computing.

The design of IASERV centers around seamless integration of such enabling technologies as ontologies, Web Services and the Foundation for Intelligent Physical Agents (FIPA)-compliant agent framework Java Agent Development Environment (JADE) [45] to provide personalized and context-aware healthcare services. The services are implemented as web services and deployed in a cloud computing environment for economic, scalable, and ubiquitously accessible service provision as illustrated in Figure 1.

**Figure 2:**
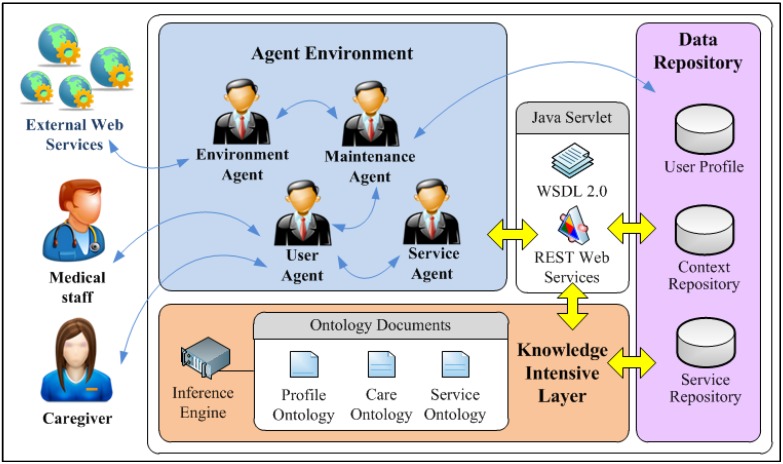
The IAServ architecture.

Functionally, the IAServ is able to infer the appropriate care plan which defines the needed healthcare activities through the patient’s profile and relevant external web services. The care-givers administrate healthcare according to a pre-set medical goal. Care-givers can also access contextually-relevant information (e.g., the impact of recent weather changes for a person suffered from hypertension) through any mobile device. As shown in Figure 2, the IAServ architecture comprises four main components: (1) the Knowledge Intensive Layer; (2) the cloud-based web services; (3) the agent environment; and (4) the Data Repository.

The Data Repository Layer stores the patient’s personal information and the web services description. The Web Services provide the web applications related to the tasks encapsulated in the care plan such as reminders, weather information, *etc.* These information sources play vital role in providing personalized and context-aware healthcare services. The agents in the Agent Environment serve as a mechanism for communication between the users and applications. They also execute the healthcare services defined in the care plan by dispatching the Web Services with real-time contextually-aware information through the HTTP protocol. The details of IAServ components are depicted as follows:

### 3.1. Knowledge Intensive Layer

The Inference Engine in the Knowledge Intensive Layer plays a vital role in deriving personalized care plan based on a patient’s profile. A health-care provider first compiles a patient’s profile into an ontological format through a predefined User Interface (UI). The ontological profile will be subsequently used as input to the Inference Engine for generating a personalized care plan by using the Care ontology and Service ontology.

**Figure 3:**
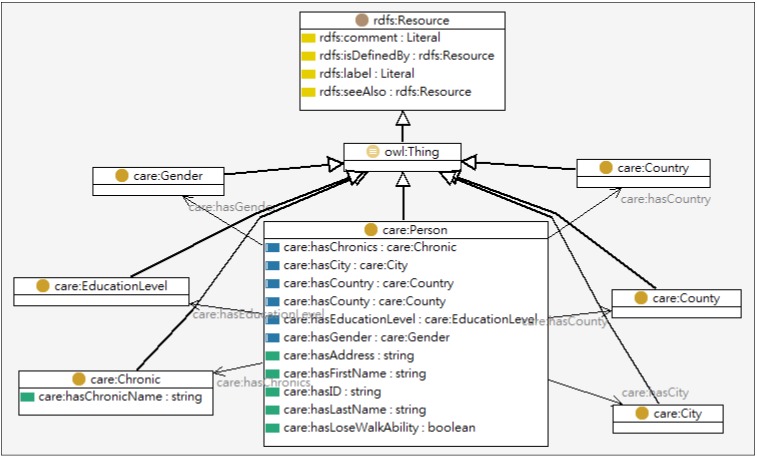
Partial model of user profile ontology.

The personal profile includes a patient’s basic information (e.g., name, sex, age, characteristics, preferences, interests, *etc.*) and personal states (e.g., clinical states, disabilities, impairments, *etc.*). Table 1 defines the classes in an ontological profile, and the partial profile ontology model is shown in Figure 3.

**Table 1:** Ontology classes of a personal profile.

Class	Annotation
Person	Basic user information (e.g., name, sex, age, *etc.*)
Characteristics	General user characteristics (e.g., height, weight, *etc.*)
Ability	User abilities and disabilities, both mental and physical
Chronic Status	User diseases (e.g., hypertension, hemorrhagic stroke)
Living Conditions	Information relevant to the user’s residence
Contact	Other persons, with whom the person is related (e.g., including relatives, friends, co-workers)
Preferences	User preferences (e.g., love cats, prefer blue color, dislike classical music)
Interests	User hobby or work-related interests. For example, “interested in sports”, “interested in cooking”
Activity	User activities, hobby or work related
Education	User education issues, including for example university diplomas and languages
Profession	The user’s profession
Expertise	computer, Internet, mobile devices, *etc.*

**Figure 4:**
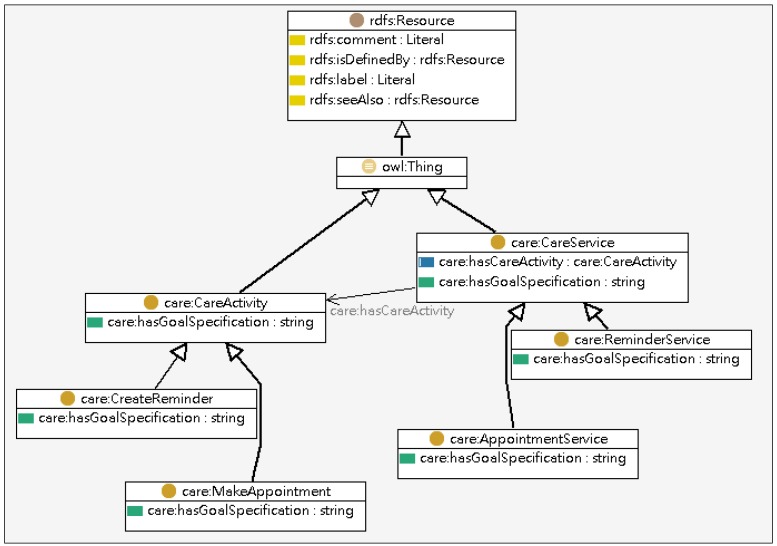
Partial model of the care ontology.

The Care Ontology encapsulates the relationship between the patient’s profile and the relevant care services. The classes defined in Care Ontology include User State, Goal-specification, Care Service, and Care Activity as illustrated in Figure 4. The User State class indicates the status of a patient while the Goal-specification and the Care Service classes describe the intended medical goal and define the care services and tasks associated with specific sates respectively.

**Figure 5:**
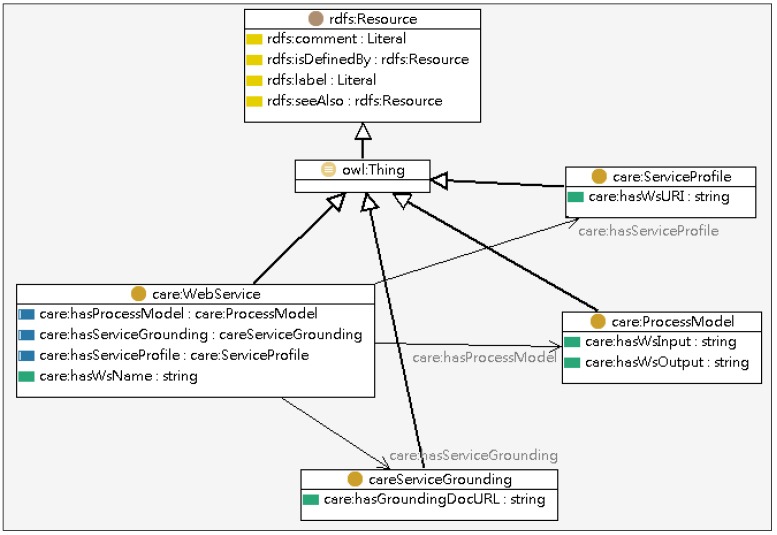
Partial model of the service ontology.

The Service Ontology encapsulates the relationship between the care activities and the physical web services. The classes covered in the Service Ontology include Care Activity, Web Service, Service Profile, Process Model, and Service Grounding, as shown in Figure 5. The Care Activity specifies the tasks that need to be performed in a care service while the Web Service registers services that are available for executing tasks.

**Figure 6:**
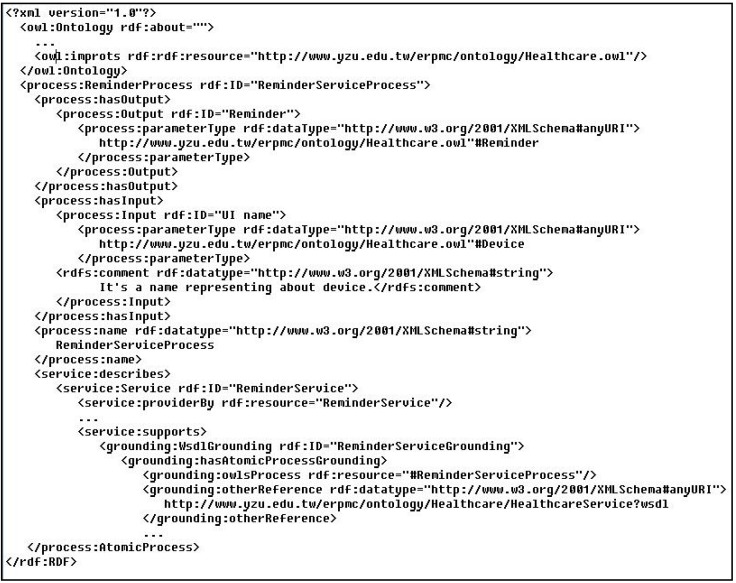
OWL-S description of the reminder service.

The OWL-S (Semantic Markup for Web Services) is adopted to describe Semantic Web Services and enable agents to automatically discover, invoke, compose, and monitor web services under specified constraints [46]. The OWL-S high level classes used in IAServ are depicted in Table 2. Figure 6 shows an OWL-S description of a reminder service as an example.

**Table 2:** High level OWL-S classes.

Class	Description
Service Profile	Includes service name and parameter annotations.
Process Model	Includes information, including input (input type), output and local.
Service Grounding	Defined by WSDL (Web Services Definition Language) 2.0 operations.

The ontologies and reasoning rules are collaboratively carried out with the help of domain experts (e.g., physicians, therapeutists, *etc.*). The Inference Engine is represented by description logic rules. Based on the interaction between the personal information and environmental context, the rules encoded knowledge furnishes inferences for dynamic situation analysis and decision making. The logic rules and constraints are defined using the SPARQL Inferencing Notation (SPIN) framework [47], which allows us to leverage the fast performance and rich expressivity of SPARQL in IAServ. An example of the rule infers a hypertension patient who lives in Tainan and needs a “Cold Wave Advice” service. The patient will be informed when the outdoor temperature drops below 13 degrees Celsius:

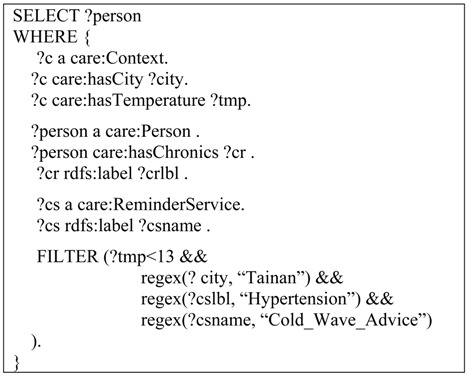



### 3.2. Web Service in the Cloud

Web services can be described as a technology and platform-independent architecture where loosely coupled components communicate via interfaces over standard web protocols. Software, hardware, and data-centric designs maximize system efficiency, scalability, and network throughput. Such web services are ideal for use in distributed systems such as IAServ where scalability is critical, both in terms of processing power and communication efficiency. The web service can be invoked by a simple HTTP query to a defined URL. The response can be HTML, comma-delimited data, XML, or a more sophisticated document type (such as a spreadsheet).

The Web Services Definition Language (WSDL) 2.0 is an XML format for describing network services as a set of endpoints operating on messages containing either document-oriented or procedure-oriented information. WSDL 2.0 describes Web services in two levels: an XML-based reusable abstract interface and concrete details regarding how and where this interface can be accessed. The Version 2.0 of WSDL features better support for REST-based web services and implementation simplification, which are essential for the development of IAServ.

The web services in IAServ are written in Java and are deployed in the Google App Engine, which provides a free environment (up to a certain level of used resources) for developing and hosting web applications in Google-managed data centers. Future work will also consider other cloud-based platforms for deployment of our web services, including Amazon Web Services [48] and Microsoft’s Azure Services Platform [49].

**Figure 7:**
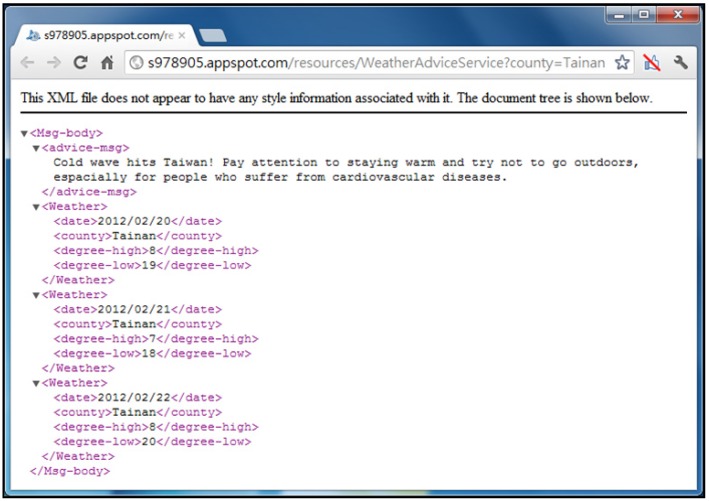
The “Weather Advice” web service in Google App engine.

**Figure 8:**
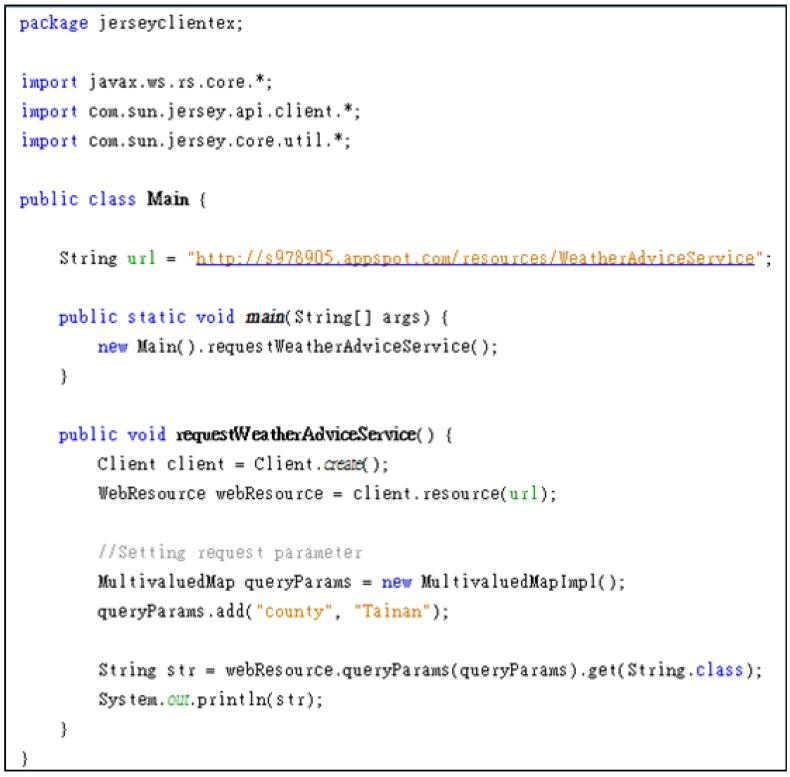
The sample Java code of accessing the “Weather Advice” web service.

Figure 7 shows an example of “Weather Advice” Web Service deployed on the Google App Engine [50]. Using simple HTTP request methods, the “Cold Wave Advice” Web Service can be easily accessed via the URL, “http://s978905.appspot.com/resources/WeatherAdviceService”, with a request parameter, “county”, and responds with an XML-formatted document. Figure 8 provides a sample Java code for accessing the Weather Advice Web Service.

### 3.3. Agent Environment

**Figure 9:**
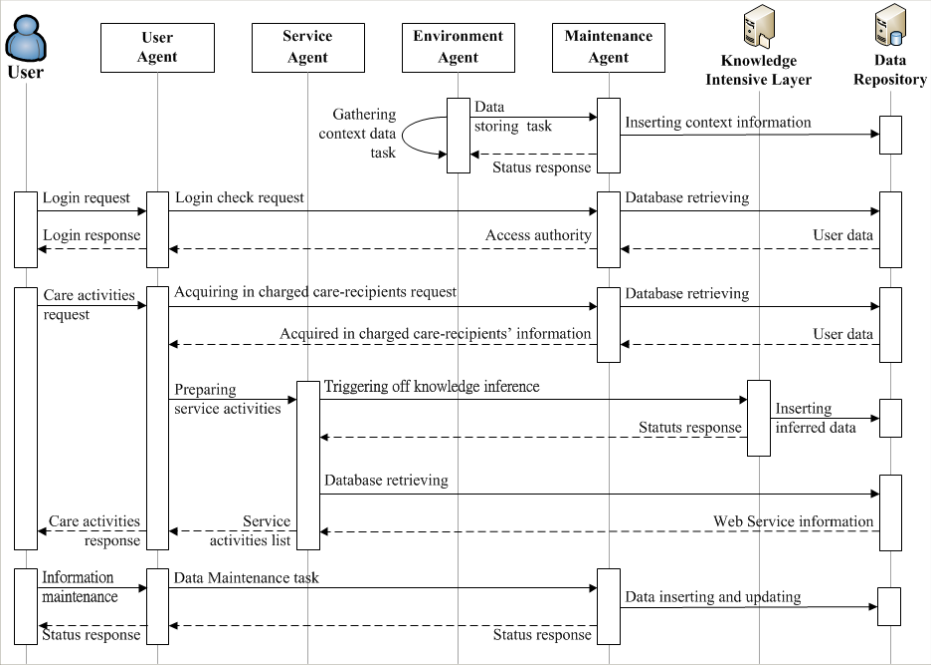
Interactions among agents, knowledge intensive layer, and data repository.

The Agent Environment is in charge of the management and coordination of all system applications and services. The agents would perform the defined care services in a care plan for a patient by dispatching the Web Services with the contextual data through the HTTP protocol. The agent environment in IAServ comprises four agents and each one of them with specific roles, capabilities and characteristics is described as follows. The interaction between the agents is illustrated in Figure 9.

**Figure 10:**
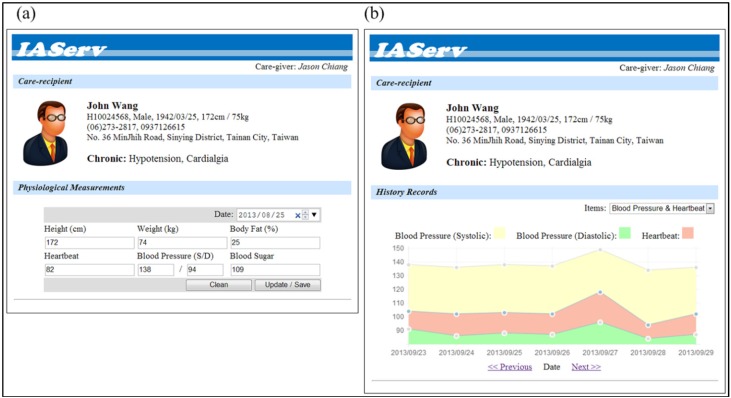
(**a**) User agent GUI for user profile submission and (**b**) for performance monitoring.

- *Environment Agent*: The Environment Agent is responsible for managing the environmental data (e.g., temperature, humidity, location, *etc.*). It works with the Context Repository and connects with the External Web Services to collect the environmental data from sources such as Yahoo! Weather [51], and stores the data into the Context Repository. When performing contextually relevant services, the Environment Agent offers the corresponding contextual data to the Service Agent for the provision of contextually-aware services.
- *Service Agent*: The Service Agent is designed to monitor the implementation of the personalized care plan. When it detects the scheduled care plan, it will use contextual data to prepare information from relevant web services and subsequently delegate task execution to the User Agent.
- *User Agent*: The User Agent is responsible for accessing the web service and performing the care services on behalf of patients when it receives tasks from the Service Agent. Following data provided by the Service Agent, the User Agent makes a request to the web service with the needed input data parameters via the HTTP protocol. The resulting information is then published to the user’s device by the User Agent to complete the care service.
- *Maintenance Agent*: Expert knowledge and patient conditions and experience may change over time. These changes can be accommodated appropriately in the ontologies through the Maintenance Agent. The Maintenance Agent plays an important role in dealing with the dynamic aspects of the health care environment. It allows experts to update the knowledge, instances, and rules encapsulated in the ontologies, as illustrated in Figure 10.

### 3.4. Data Repository

The Data Repository serves both as storage for the User Profile, Context, and Services and as a central processing point for the requests of all data. The User Profile encapsulates general information regarding care-givers and patients, which is governed by medical staff. A care-giver’s profile comprises personal information, qualifications, track records, and experiences while a patient’s profile contains his/her demographics, location, contact information, and services prescribed/subscribed.

The Context Repository stores the contextual ontology, instances (e.g., temperature, humidity, air quality) and rules, and allows care-givers to query or manage contextualized knowledge. The Service Repository stores the description of available web services including service features, inputs, outputs, locations (URI), *etc.*, and allows care-givers to manage the services.

## 4. Implementation

### 4.1. Hardware and Software Configuration

The IAServ is implemented on the Windows™ platform. To derive personalized a care plan based on a user profile, an inference engine is needed to take an “asserted” model as input and create an “inferred” model as output in the context of ontology. The past decade has seen the development of several inference engines including OWL DL tableaux reasoners like Pellet, Flora-2 implemented F-OWL, RacerPro, FacT++, Minerva, and Rule engines such as Jena [52]. Most implementation require the integration of various inference engines. For instance, the system requires running SPARQL or Jena which have no built-in knowledge of OWL DL on top of an OWL-compliant reasoning service such as Pellet. Considering potential future expansion of IAServ, we adopted TopBraid™ Composer (TBC) [53] as our development platform. The Eclipse-based TBC is not only a visual ontology editor but also a knowledge-based framework capable of integrating such built-in inference engines, as TopSPIN [54], Pellet, Jenna, *etc.* through hybrid inference-chaining. TopSPIN, the embedded rule-based reasoner in TBC, which supports SPARQL query and SPIN rules, is used as the main inference engine in IAServ. Its built-in ability to run multiple inference engines shortens the IAServ development cycle.

The Agent Environment in IAServ is implemented in the FIPA-compliant JADE framework. To run JADE, we need to install the Java 2 Run-time Environment (JRE). However, we recommended installing the full Java 2 Source Development Kit (J2SDK) because it is used in the development and compilation of our own agents in JADE.

### 4.2. Usage Scenario and Demonstration

The IAServ allows healthcare professionals to administer a personalized service plan and subscribe to needed services in the healthcare context by using practically any Internet-enabled device. In this section, we describe an implementation scenario to illustrate IAServ usage. “John Wang is 72 years old. Despite his age and having various medical conditions, he prefers to remain in his own home. John suffered a hemorrhagic stroke with mild claudication and hypertension, and needs to go to the hospital three times a week (Monday, Wednesday, and Thursday every week at 9:30 A.M.) for rehabilitation and to take blood pressure medicine twice daily. However, John was difficulty walking to the hospital because of claudication, and frequently forgets to take his medicine. In John’s case, the care-givers may use IAServ to provide personalized healthcare plan to address these problems.” [55].

**Figure 11:**
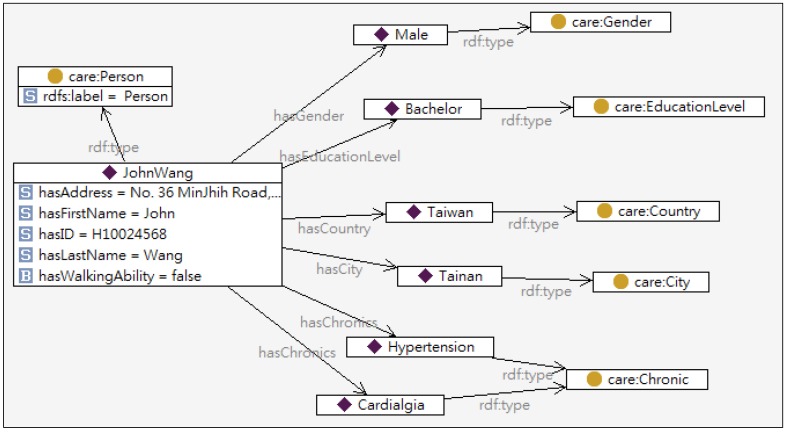
Partial ontology model of John’s profile.

To produce a personalized healthcare plan which fulfills John’s desirable goal “remain living in his own home”, a healthcare professional first submits John’s profile to IAServ via the Maintenance Agent’s GUI, as shown in Figure 10. Figure 10a shows the GUI for care-giver to record basic physiological signals, and the history records are also provided for observation and monitoring of the patient’s performance, as shown in Figure 10b. The profile is subsequently converted into an ontology specification as depicted in Figure 11 and stored in the personal information repository. The inference engine then generates a personalized care plan for John based on the CARE ontology and Service ontology.

**Figure 12:**
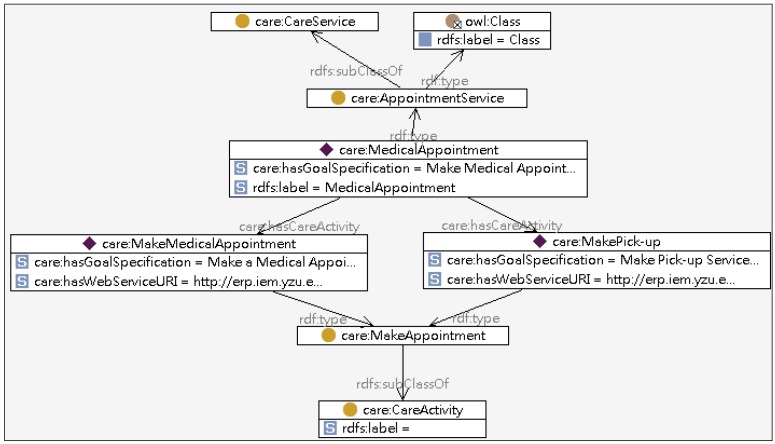
The partial ontology model of “Medical Appointment Service”.

Given John’s clinical state of “Hemorrhagic Stroke”, the inference engine generates a relevant care service “Medical Appointment Service” based on the Care Ontology, which will be included in John’s care plan. Similarly, the inference engine infers the clinical state “Hypertension”. The generated care services “Reminder Service” and “Weather Service” will also be included in John’s care plan. The partial Care Ontology is shown in Figure 12.

**Figure 13:**
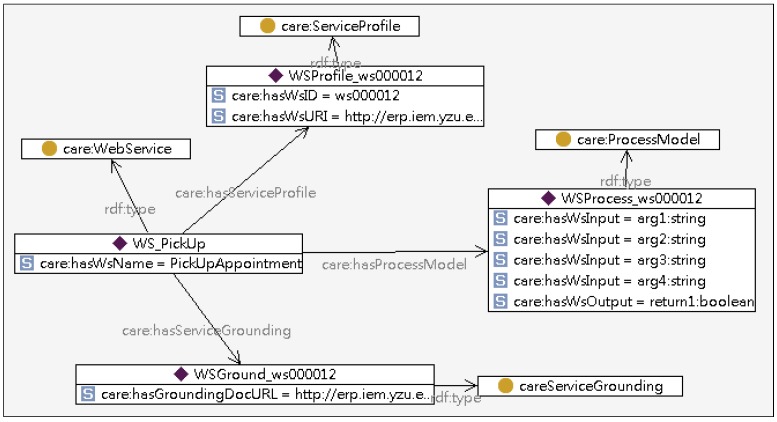
Partial ontology model of “Pick-up Web Service”.

If the generated care plan is approved by John’s care-giver, the tasks required to complete the services in the plan will be further inferred based on the Service ontology as shown in Figure 13. In this example, the “Medical Appointment Service” may consist of “Appointment Making” and “Round-trip Pickup” (as John suffers from claudication) to aid John in making the appointment. A “Voice Reminder” activity using an event-driven calendar may be generated to provide a “Reminder Service” for John to take his medicine. If John’s profile indicates he has hearing problems, the reminders can automatically be produced as text-messages or video-messages. Weather has a significant impact on blood pressure. Because John suffers from hypertension, changes in weather will automatically trigger appropriate reminders for John to closely monitor his blood pressure.

**Figure 14:**
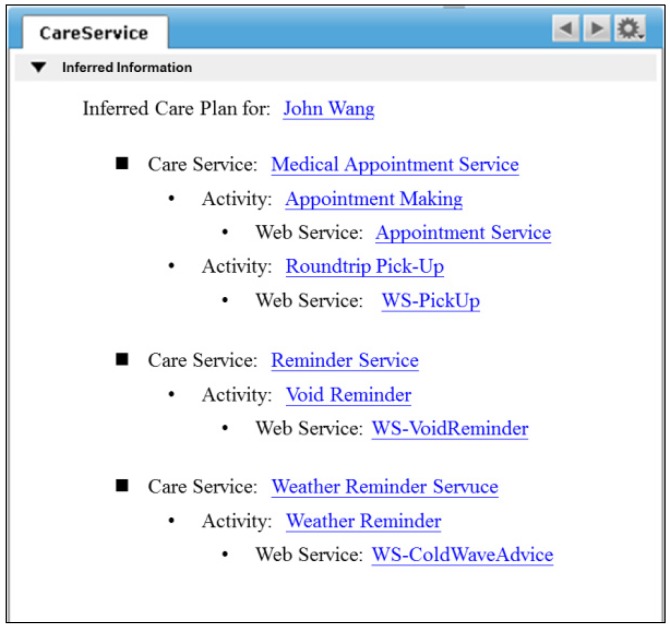
Inferred information page of care services.

Once the services and related tasks are inferred, a care plan is generated and stored in the personal information repository as shown in Figure 14. The web services associated with the tasks can be subscribed for plan execution.

**Figure 15:**
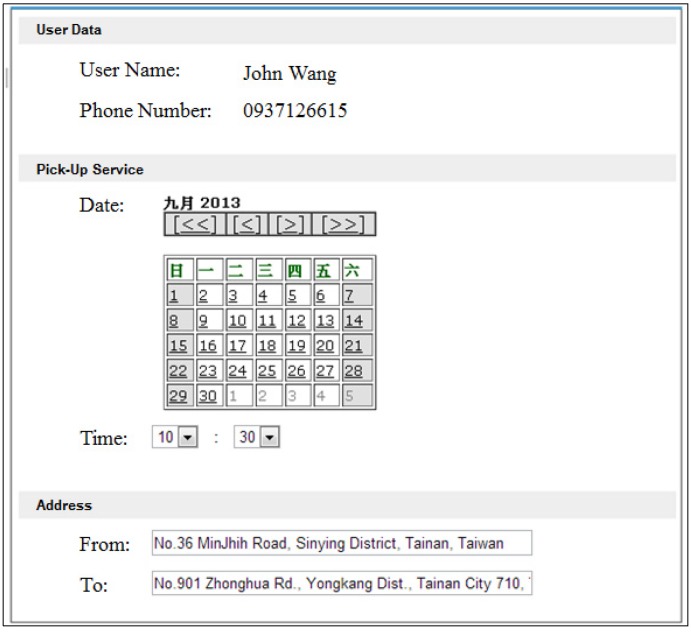
Pick-up web service settings form.

John’s care plan may be adjusted periodically by his physician, depending on his health conditions or the outcome of plan execution. For example, if arranging pick-up service for John on Monday, Wednesday, and Thursday at 9:30 A.M. every week can be accomplished using a built-in GUI, as illustrated in Figure 15.

**Figure 16:**
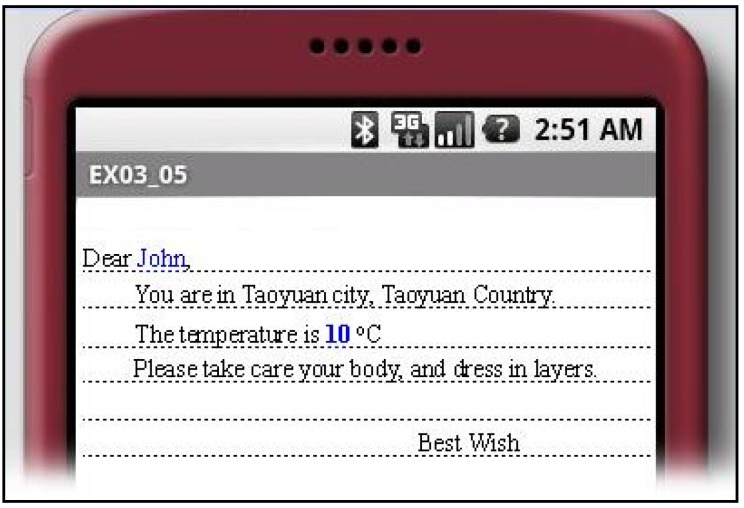
Example of cold wave adviser service message.

The web services are implemented in Java using a REST-style architecture and deployed in a cloud environment. The services can therefore be reliably accessed from anywhere on virtually any Internet-enabled device, as shown in Figure 16.

### 4.3. System Usability Evaluation

System usability/readability was evaluated through a questionnaire distributed to twenty care-givers and twenty patient users. Their respective profiles are illustrated in Table 3 and Table 4.

**Table 3:** User profiles (care-givers).

Personal information	Number	%
*Age*		
20–30	4	20.0
30–40	13	65.0
40 or above	3	15.0
Total	20	100.0
*Gender*		
Male	8	40.0
Female	12	60.0
Total	20	100.0
*Service year*		
1 or below	6	30.0
1–3	7	35.0
3–5	6	30.0
5 or above	1	5.0
Total	20	100.0
*Experience of using Internet*		
Yes	15	75.0
No	5	25.0
Total	20	100.0
*Experience of using Smart Mobile Device*		
Yes	11	55.0
No	9	45.0
Total	20	100.0

**Table 4:** User profiles (patients).

Personal information	Number	%
*Age*		
45–55	10	50.0
55–65	8	40.0
65 or above	2	10.0
Total	20	100.0
*Gender*		
Male	8	40.0
Female	12	60.0
Total	20	100.0
*Patient with numbers of chronic disease*		
0	4	20.0
1–2	11	55.0
3 or above	5	25.0
Total	20	100.0

**Figure 17:**
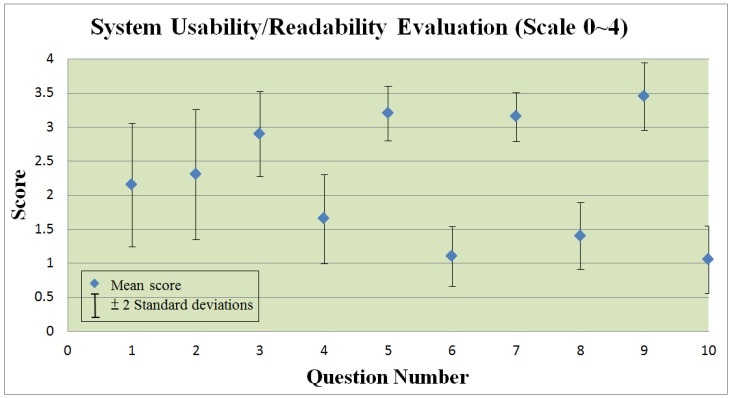
System usability/readability evaluation for health-care professional users.

**Figure 18:**
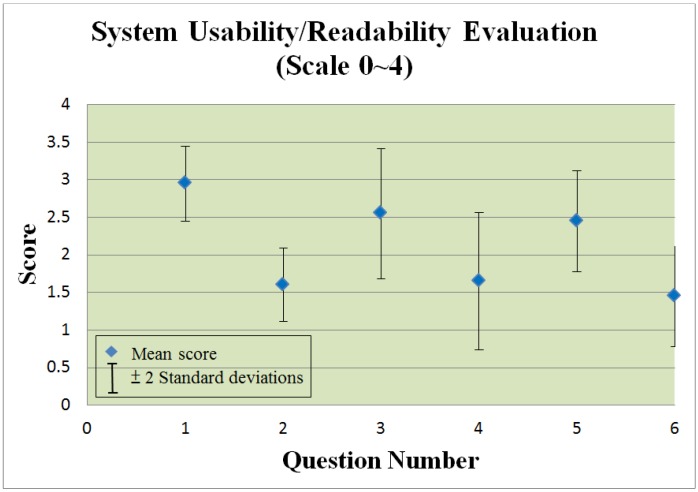
System usability/readability evaluation for patients.

Figure 17 and Figure 18 present a statistical summary of usability/readability respectively obtained from the questionnaires for professional and general users. For each section, the mean score and a range representing +/−2 standard deviations is displayed.

In our questionnaire, question validity is reinforced by posing each question twice: once as a positive statement and followed by a negative statement The figure indicates that the usability/readability of the IAServ is reasonably high. The question numbers below correspond to the x-axis in Figure 17:

1. IAServ was easy to use.
2. I would need a technical person to assist me.
3. IAServ is useful in providing better services to patients.
4. IAServ wasn’t useful in improving service quality.
5. IAServ significantly reducing the workload of care-givers.
6. The system did not significantly assist care-givers.
7. The features offered by IAServ are sufficient.
8. The features offered by IAServ are insufficient.
9. IAServ significantly contributes to improving the delivery of health care services.
10. Care-givers won’t find IAServ isn’ helpful in serving patients.

The question numbers below correspond to the x-axis in Figure 18:

1. I would like to offer health care services using IAServ.
2. I’m not interested in using the IAServ platform.
3. The features offered by IAServ are sufficient.
4. The features offered by IAServ are insufficient.
5. IAServ was useful in helping to maintain my health.
6. IAServ was useless for managing my health.

## 5. Concluding Remarks and Future Work

Personalized and context-aware healthcare services can be provided through proposed IAServ system, based on seamless integration of such enabling technologies as Ontology, Web Services and the Foundation for Intelligent Physical Agents (FIPA)-compliant agent framework Java Agent Development Environment (JADE). The IAServ employs ontologies to improve the integration and orchestration of all the technologies involved such as web services, context-aware healthcare services, and software agents. Rules of inference are formulated, using structural information in ontologies to derive personalized, context-aware healthcare plan for elderly patients. The proposed system design can serve as the foundation for an economic, scalable, and robust Web-based healthcare platform. Table 5 presents a comparison with similar studies discussed in Section 2 to highlight the advantages and contributions of the proposed IAServ. While the comparison might not be comprehensive, it provides an overview of the differences between the IAServ and our understanding of other similar systems.

**Table 5:** Comparison of IAServ and other proposed systems.

Reference	Service domain	Ambient intelligence	Multi-agent system	Context-aware reasoning	Web services based architecture
Riva, 2003 [12]	Smart home environment	Yes	No	No	No
Su and Chiang, 2013 [20]	Home health care	Yes	Yes	Yes	No
Coronato *et al.*, 2009 [21]	Smart hospital environment	No	No	Yes	No
Fraile *et al.*, 2009 [22]	In-home Monitoring	Yes	Yes	Yes	No
Eslami *et al.*, 2010 [27]	Care service tailoring	Yes	No	Yes	No
Chang *et al.*, 2009 [28]	In-home Monitoring	Yes	No	No	No
Fenza *et al.*, 2012 [23]	In-home Monitoring	Yes	Yes	Yes	No
IAServ	Home health care	Yes	Yes	Yes	Yes

One critical issue yet to be addressed is the protection of user privacy and personal information. While the advantage of mobility in the agent paradigm helps us in developing distributed health care systems and reaching a larger mobile clientele, it also leaves the system vulnerable to attacks by malicious entities. A JADE plug-in (JADE-S [56]) was to provide security through access controls and secure communications, making the platform suitable for use in real environments. While JADE-S provides fundamental support for application-level security, it suffers from several shortcomings. For example, as SSL must be applied globally to all messages, and agent identity information cannot be accessed from the program. User data can be better protected by incorporating more sophisticated and robust security models in the IAServ server [57,58,59].
